# Simultaneous Health Risk Assessment of Potentially Toxic Elements in Soils and Sediments of the Guishui River Basin, Beijing

**DOI:** 10.3390/ijerph16224539

**Published:** 2019-11-16

**Authors:** Jiankang Wang, Bo Gao, Shuhua Yin, Dongyu Xu, Laisheng Liu, Yanyan Li

**Affiliations:** 1 State Key Laboratory of Simulation and Regulation of Water Cycle in River Basin, China Institute of Water Resources and Hydropower Research, Beijing 100038, China; shuibenerban@126.com; 2 Department of Water Environment, China Institute of Water Resources and Hydropower Research, Beijing 100038, China; yinsh@iwhr.com (S.Y.); xudy@iwhr.com (D.X.); Liuls112@126.com (L.L.); lyy2018_ts@163.com (Y.L.)

**Keywords:** soil, sediment, potentially toxic elements, health risk assessment, Guishui River

## Abstract

Simultaneous ecological and health risk assessments of potentially toxic elements in soils and sediments can provide substantial information on their environmental influence at the river-basin scale. Herein, soil and sediment samples were collected from the Guishui River basin to evaluate the pollution situation and the ecological and health risk of potentially toxic elements. Various indexes were utilized for quantitatively assessing their health risks. Pollution assessment by geo-accumulation index showed that Cd had “uncontaminated to moderately polluted” status in the soils and sediments. Potential ecological risk index showed that the Guishui River basin was at low risk in general, but Cd was classified as “moderate or considerable ecological risk” both in the soils and sediments. Health risk assessment calculated human exposure from soils and indicated that both non-carcinogenic and carcinogenic risks of the selected potentially toxic elements were lower than the acceptable levels. Health risks posed by potentially toxic elements bio-accumulated in fish, stemming from sediment resuspension, were also assessed. Non-carcinogenic hazard index indicated no adverse health effects on humans via exposure to sediments; however, in general, Cr contributed largely to health risks among the selected potentially toxic elements. Therefore, special attention needs to be paid to the Guishui River basin in the future.

## 1. Introduction

Potentially toxic elements (PTEs) are among the most ubiquitous pollutants found in the environment [1]. The obvious threat that comes from PTEs is accumulation in the environment since they are refractory and carcinogenic [2]. With the acceleration of industrialization, urbanization, and agriculture, soil and sediment pollution by PTEs has accelerated in China in the last two decades [3,4]. Especially at a river basin scale, PTEs in soil and sediment can undergo migration and transfer though natural processes or anthropogenic activities [5,6]. The high toxicity and wide prevalence of PTEs in the soils and sediments has been a potential risk for the environment and human health [7]. For example, humans can be directly exposed to contamination from soils by ingestion of, inhalation of, and dermal contact with soil particles [8,9,10]. Meanwhile, humans will be at risk from sediments indirectly in cases of intake of the PTEs through aquatic food products (e.g., fish and scallops) [11,12]. Hence, a better understanding of the ecological impacts and health risks of PTEs in soils and sediments of the river basin is worthwhile.

At present, for assessing the soil and sediment contamination in river basins, the geo-accumulation (*I_geo_*) and potential ecological risk indices (*RI*) are the most conventional techniques, which are used to evaluate the possibility of a strong ecological impact because of exposure to the environment [13,14]. For assessing the health risk to humans, two indexes that were proposed by the US Environmental Protection Agency (USEPA) [15], can be calculated: non-carcinogenic and carcinogenic risks [16]. Previous studies, however, have mostly focused on the health risk evaluation of a single medium, such as exposure from polluted soil particles [10,17] or from aquatic products consumption [18]. Simultaneous evaluation of the human health risks posed by the PTEs in soils and sediments at the scale of a river basin is infrequent, and the evaluation of a single medium within a river basin may not comprehensively cover the level of PTEs pollution. Thus, in this study, to comprehensively evaluate the health risks to humans, a new bioaccumulation assessment model was applied to estimate PTEs accumulation in fish, and the non-carcinogenic risks during sediment disruption were then calculated [19,20]. In addition, comparing the health risk assessments of humans under two different pathways (sediment and soil) is helpful for defining of guidelines or screening the levels of soil and sediment contamination.

**Figure 1:**
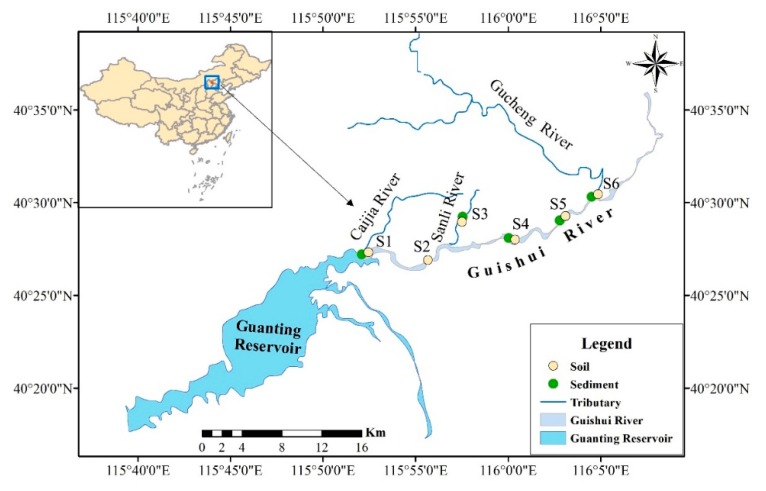
Location of the sampling sites of soils and sediments of the Guishui River basin.

Guishui River (40°26′–40°29′ N, 115°52′–116°05′ E) is located in the Yanqing County, northern Beijing (Figure 1). It is the venue for the World Horticultural Exposition (2019) and the International Winter Olympics (2022). The Guishui River originates in Yanqing County, Beijing, and flows into the Guanting Reservoir. The total length and area of the Guishui River is 18.5 km and 1073.6 km^2^, respectively. The environmental quality of the Guishui River in Yanqing country has received extensive attention. In this study, the main goals were to: (1) investigate the pollution status of PTEs (As, Cd, Cr, Co, Cu, Ni, Pb, V and Zn) in the soils and sediments of the Guishui River basin; (2) evaluate the human health risks of selected PTEs via exposure to soils; and (3) estimate the enrichment of individual PTEs in fish and assess the health risks of PTEs in sediments to humans, while discussing the difference in the health risk between exposure to soils and exposure to sediments.

## 2. Materials and Methods

### 2.1. Sample Collection

In this study, five superficial sediment samples (0–10 cm in depth and approximately 2 kg) and six soil samples (0–10 cm in depth and approximately 2 kg) were collected in the Guishui River basin (Figure 1) in May 2018. At each sampling site, three replications were collected and homogenized after separation to form a composite sample. All the collected samples were kept in polyethylene bags and sealed. After sampling, the samples were transported to the laboratory and frozen, lyophilized at −80 °C, ground in an agate mortar, and passed through a 100-mesh nylon sieve for total metal analysis.

### 2.2. Analytical Methods

The total metal concentrations in the soils and sediments were performed according to Gao et al. [21]. First, each sample (0.04 g) were weighed and dissolved into 10 mL Teflon bombs, then 2 mL 65% HNO_3_ and 0.2 mL 30% H_2_O_2_ were sequentially added. In this step, the samples were left on a hot plate for 24 h for removing the organic matter. The residue samples were dried at 120 °C. Secondly, 1 mL 65% HNO3 + 1 mL 40% HF were sequentially added, and the samples were then transferred to sealed bombs and placed in an oven at 190 °C for 48 h. In the second step, clear solutions were obtained. Thirdly, the samples were evaporated at 120 °C. Finally, the samples were subjected to ultrasound treatment for 30 min and dissolved in 1% HNO3 (v:v). The concentrations of PTEs were measured by inductively coupled plasma mass spectrometry (Elan DRC-e, PerkinElmer, Waltham, MA, USA).

Quality control was performed using certified reference material of stream sediment (GSD–1a, GBW07301a), produced by the Institute of Geophysical and Geochemical Exploration, Chinese Academy of Geological Sciences. Reagent blanks, a standard reference material, and sample replicates were used to assess the accuracy and precision of the analyses. We obtained good agreement with the reference values (Table S1). All of the reagents were guaranteed analytical grade or higher. The laboratory glassware (bottles, tubes, etc.) were pre-cleaned by soaking in 20% HNO_3_ (*v*/*v*) for at least 24 h, followed by soaking and rinsing with deionized water before use.

### 2.3. Assessment Methods

#### 2.3.1. Geo-Accumulation Index (*I_geo_*)

The *I_geo_* was first introduced by Müller [22] and is the most widely used index for evaluating metal contamination in soils and sediments. The calculation formula is given in Equation (1):

(1) $$I_{geo}=\log_{2}\left(\frac{C_{i}}{1.5B_{i}}\right)$$

where *C_i_* is the measured concentration of a PTEs element in the sediment, *B_i_* represents the background values in the soil of Beijing [23], and 1.5 is the background matrix correction factor. The relationship between *I_geo_* and the pollution level is presented in Table S2 using 7 classes from 0 to 6 to indicate the pollution status.

#### 2.3.2. Potential Ecological Risk Index (*RI*)

The *RI* was introduced by Häkanson [24] to evaluate the potential ecological risk of PTEs in soils and sediments. Multiple influencing factors (ecological sensitivity, synergy, and toxicity level) are comprehensively considered in this method. The potential ecological risk (*EI*) factor is calculated as given in Equation (2):

(2) $$E_{i}^{}=T_{i}^{}\times \frac{C_{i}}{C_{0}^{}}$$

Equation (3) was used to calculate the RI of sampling sites as follows:

(3) $$RI=\sum_{i=1}^{n}\left(T_{i}^{}\times \frac{C_{i}}{C_{0}}\right)$$

where $C_{i}$ is the measured concentration of PTEs in the sediment/soil, $C_{0}$ is the reference background value, and $T_{i}^{}$ is the toxic-response factor of an individual element, which was determined to Cu = Pb = Ni = 5, Zn = 1, As =10, Cr = 2, and Cd = 30 [24,25,26,27]. *EI* is the potential ecological risk factor of a single metal, and *RI* is the potential ecological risk index of the PTEs. The degrees of *EI* and *RI* classification are presented in Table S3.

### 2.4. Health Risk Assessment in the Guishui River Basin

#### 2.4.1. Health Risk Assessment of Potentially Toxic Elements in the Soil

The health risk assessment model was developed by the U.S. Environmental Protection Agency [15], and was applied to calculate human exposure to PTEs from soils. The calculation results of the model can be used as the definition of guidelines or screening levels of soil contaminants. PTEs in the surface soil enter the human body through three main pathways: ingestion (*D_ing_*), inhalation (*D_inh_*), and dermal contact with soil particles (*D_dermal_*).

The average daily dose contacted through each of the three pathways was calculated using the following Equations (4)–(6) [15,28]:

(4) $$D_{ing}=C\times \frac{IngR\times EF\times ED}{BW\times AT}\times 10^{-6}$$

(5) $$D_{inh}=C\times \frac{InhR\times EF\times ED}{BW\times AT\times PEF}$$

(6) $$D_{dermal}=C\times \frac{SL\times SA\times ABS\times EF\times ED}{BW\times AT}\times 10^{-6}$$

The definition of each parameter is presented in Table S4.

For the non-carcinogenic risk, hazard quotients (*HQ_i_*) were calculated using Equation (7). The hazard index (*HI*) was equal to the sum of *HQ_i_*. If the *HI* < 1, the daily exposure is unlikely to cause adverse health effects; but if the *HI* > 1, it will cause possible adverse health effects [29]. *HI_soil_* (the *HI* that was calculated through the exposure to soils) was applied to assess the overall non-carcinogenic risk which was calculated using Equation (8). For the carcinogenic risk (*CR*), due to the lack of reference doses for evaluating the dermal and ingestion absorption, the *CR* was calculated using the reference doses of inhalation, and was shown in Equation (9) [30]:

(7) $$HQ_{i}=\frac{D_{i}}{R_{f}D_{i}}$$

(8) $$HI_{soil}=\sum HQ_{i}$$

(9) CR=D×SF

The definition of each parameter is presented in Table S3.

#### 2.4.2. Health Risk Assessment of Potentially Toxic Elements in the Sediment

The health risk assessment of PTEs in sediment is mainly estimated by the PTEs released through the sediment disturbance process, which are absorbed by aquatic organisms and then enter the humans through an intake route. Thus, in this study, the concentrations of seven selected PTEs (Cd, Cr, Cu, Ni, Pb, V, and Zn) in fish were assessed by using the environmental impact assessment model which is a hypothetical model [19]. The assessment model, without using any real measurements in fish, was successfully applied to evaluate the health risks of PTEs during the sediment resuspension [18,31]. The PTEs concentrations in fish (*C_f_*) are calculated as shown in Equation (10):

(10) $$C_{f}=BCF\times C_{w}$$

The definitions of *C_f_*, *BCF*, and *C_w_* are shown in Table S4.

In aquatic environments, the total PTEs concentration (*C_t_*) desorbed from the solid phase (mostly existing in the total suspended solids (TSS)) is calculated using Equations (11) and (12):

(11) $$C_{t}=TSS\times C_{s}$$

(12) $$C_{t}=C_{w}+TSS\times C_{p}=C_{w}+TSS\times K_{d}\times C_{w}$$

The definitions of *C_t_*, *TSS*, *C_s_*, *C_p_*, *C_w_*, and *K_d_* are also shown in Table S4.

According to the results of *C_t_*, the health risk assessment of PTEs in sediment was calculated by the *HQ_i_*, which is in accordance with the guidelines of the USEPA Region III risk-based concentration table [32]. The *HQ_i_* is calculated using Equations (4) and (7), as mentioned above. Due to ingestion being the main pathway for the fish PTEs to enter human bodies, the *HI_sediment_* (the *HI* that was calculated through exposure to sediment) was equal to the *HQ_i_* in this assessment model.

### 2.5. Statistical Analysis

Data and graphics processing were performed using SPSS 22.0 (IBM, New York, NY, USA) and Origin 2017 (OriginLab Corporation, Northampton, MA, USA) for Windows, and spatial mapping was performed using ArcGIS 10.1 (ESRI, California, CA, USA) for Windows.

## 3. Results and Discussion

### 3.1. The Concentrations of Potentially Toxic Element in the Guishui River Basin

The concentration range and average value of PTEs in soils and sediments of the Guishui River are shown in Table 1. As shown in Table 1, the average PTE concentrations in the soils were 8.57, 0.16, 52.04, 10.44, 20.04, 23.24, 25.25, 67.77, and 75.17 mg/kg for As, Cd, Cr, Co, Cu, Ni, Pb, V, and Zn, respectively. The average PTE concentrations followed the order of Zn > V > Cr > Pb > Ni > Cu > Co > As > Cd. The average PTE concentrations in sediments were 6.81, 0.14, 50.45, 10.48, 17.95, 21.78, 22.42, 66.95, and 66.76 mg/kg for As, Cd, Cr, Co, Cu, Ni, Pb, V, and Zn, respectively, followed by the sequence of V ≈ Zn > Cr > Pb > Ni > Cu > Co > As > Cd. Obviously, the mean concentrations of PTEs in the sediments were similar to those in soils, and the order of PTE content in sediments was almost the same to that in soils. Compared with the background value in the soil of Beijing [23], the average concentration of all the studied PTEs in soils and sediments were lower than the background value, except for Cd. The average concentration of Cd in soils and sediments was 3 times and 2.6 times higher than in the background value, respectively, indicating that there may be a potential risk for Cd in the Guishui River basin.

### 3.2. Pollution Assessment of Potentially Toxic Elements in the Soils

#### 3.2.1. Geo-Accumulation Index

**Figure 2:**
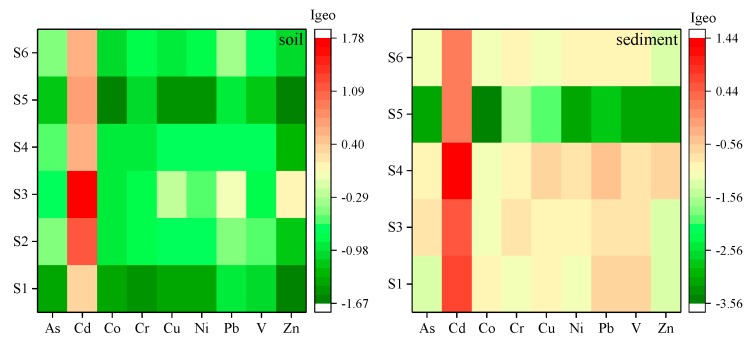
Hotmaps of *I_geo_* for potentially toxic elements in soils and sediments of the Guishui River basin.

The calculated *I_geo_* value for each of the PTEs is presented in Figure 2. In general, the average *I_geo_* values of the studied PTEs, excluding Cd, were less than zero. The mean values were −0.76 for As, 0.89 for Cd, −1.13 for Co, −0.96 for Cr, −0.85 for Cu, −0.90 for Ni, −0.59 for Pb, −0.79 for V, and −1.08 for Zn. The results indicated that soils in the Guishui River were unpolluted by the studied PTEs (except for Cd). Cd qualified for class 1, indicating uncontaminated to moderately contaminated pollution levels.

#### 3.2.2. Potential Ecological Risk Index

**Figure 3:**
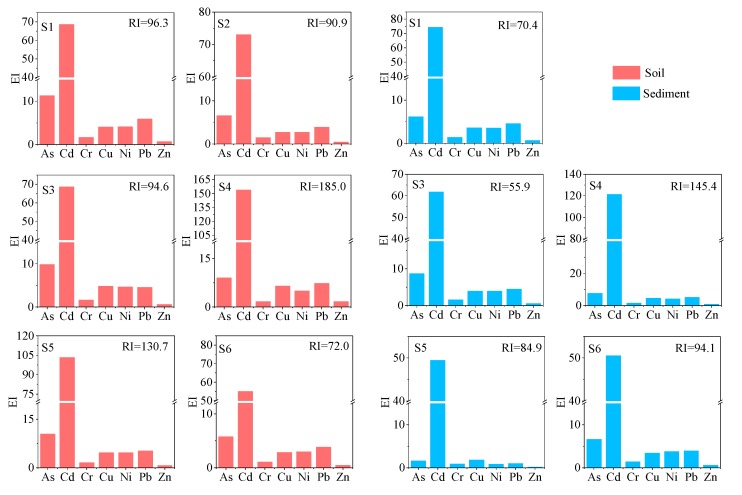
Ecological risk (EI) and *RI* values for Potentially toxic elements (PTEs) in soils and sediments of the Guishui River basin.

The *RI* value at each sampling site and the *EI* value of each of the studied PTEs are shown in Figure 3. Overall, all sampling sites were classified as low risk according to the results of RI. Specifically, the *RI* value of S4 was 185.0, indicating that the site was moderately polluted; however, the soils were generally contaminated by Cd according to the *EI* value (*EI* > 40). Previous research has proposed that the pollution of Cd may originate from the abuse of phosphate fertilizer in agriculture [33]. The Guishui River basin is dominated by agricultural growing areas (e.g., corn, vegetables and fruit trees) [34,35], especially at the S4 sampling site. Thus, it can be inferred that Cd was the predominate contaminant in the soil of the Guishui River.

#### 3.2.3. Health Risk Assessment of Potentially Toxic Element Exposure from Soils

As shown in Table 2, for non-carcinogenic effects, ingestion appeared to be the main intake route for PTEs in soils that were harmful to children, followed by dermal contact. Meanwhile, ingestion and dermal contact were both the primary exposure pathway for adults. This result is also consistent with the results of the previous studies [36,37]. Inhalation was considered to be the lowest exposure risk, which was two to five orders of magnitude lower than the other two exposure pathways. In fact, HQ values for adults were approximately one order of magnitude lower than those for children in terms of most PTE exposure to soils, excluding As and Cd (Table 2), indicating that children faced more potential harmful health risks from exposure to soils in the Guishui River basin. This is mainly due to the unique physiological characteristics, such as more frequent hand-to-mouth activities, which are often regarded as one of the critical pathways for exposure to soil PTEs in children [38]. Overall, the *HI_soil_* values of the studied PTEs were all lower than 1 (i.e., at safe levels), indicating that there were no non-carcinogenic risks from these PTEs for children or adults.

Considering the non-carcinogenic effects for adults, the *HI_soil_* value of the studied PTEs followed the order of As > Cr > V > Pb> Ni > Co > Cd > Cu > Zn; As had the highest risk value (1.58 × 10^−1^), followed by Cr (7.43 × 10^−2^), and Zn was the lowest (4.83 × 10^−4^). Compared to adults, the *HI_soil_* value for children followed the order of As > Cr > V > Pb > Ni > Co > Cu > Zn > Cd, which was similar to that for adults. It can be seen that as is the main PTE affecting the health of local residents (accounting for approximately 44.5% for children and 49.3% for adults of total *HI_soil_*), followed by Cr (21.9% for children and 20.4% for adults). It has been reported that the daily intake of As-contaminated or Cr-contaminated water or other substances respectively causes a decreases in the generation of white and red blood cells, and causes damage to the liver [32,39]. Thus, reducing the exposure frequency or ingestion rate of local residents is beneficial for mitigating potential health risks.

For the carcinogenic risk effects, As, Cd, Co, Cr and Ni were assessed through the ingestion exposure modes for soils. As shown in Table 3, the values of the carcinogenic risk were 1.13 × 10^−12^ (As), 3.08 × 10^−11^ (Cd), 6.59 × 10^−8^ (Cr), 3.12 × 10^−9^ (Co) and 5.92 × 10^−10^ (Ni) for children and 3.19 × 10^−12^ (As), 8.69 × 10^−11^ (Cd), 1.86 × 10^−7^ (Cr), 8.81 × 10^−9^ (Co), and 1.67 × 10^−9^ (Ni) for adults. Clearly, the carcinogenic risk levels for these studied PTEs were all lower than 10^−6^ magnitude (the internationally accepted precautionary criterion) [40], which indicated that the carcinogenic risk of As, Cd, Co, Cr, and Ni due to soil exposure was acceptable in the Guishui River basin. Notedly, the Cr contributed more than 94.6% to the overall carcinogenic risk for children and adults, indicating that the carcinogenic risk of Cr needs greater attention in the future.

Overall, the results showed that the risk values of both non-carcinogenic and carcinogenic materials would not cause serious health impacts in the study areas; however, it should be noted that the risk values of both non-carcinogenic and carcinogenic materials obtained in this study were based on an idealized model. Thus, there is a high degree of uncertainty about the calculated risk of both non-carcinogenic and carcinogenic materials due to PTEs exposure from soils [41]. On the one hand, there was a lack of exposure parameters and metal toxicity data; on the other hand, other PTEs (i.e., Hg, Sn, and Mn) and other potential exposure pathways (e.g., atmospheric deposition or street dusts) were not considered in this study. Even so, the health risk assessment has proven to be a useful tool in assessing the health risk of PTEs exposure from soils.

### 3.3. Pollution Assessment of Potentially Toxic Elements in the Sediments

#### 3.3.1. Geo-Accumulation Index

The calculated *I_geo_* value for each metal is presented in Figure 2. Clearly, the average *I_geo_* values of most PTEs were less than zero (except Cd), and, the mean values were −1.49 for As, 0.58 for Cd, −1.59 for Co, −1.13 for Cr, −1.17 for Cu, −1.39 for Ni, −1.16 for Pb, −1.27 for V, and −1.54 for Zn in sediments. It can be shown that there was slight Cd pollution in the sediment of the Guishui River.

#### 3.3.2. Potential Ecological Risk Index

The *RI* value at each sampling site and the *EI* value of each of the studied PTEs are shown in Figure 3. Overall, all the sampling sites were classified as low risk according to the results of *RI*. Meanwhile, according to the results of *EI*, Cd was classified as a moderate ecological risk or considerable ecological risk. In view of the risk assessment results of the soil, Cd was inferred to be the predominate contaminant in the Guishui River basin. Thus, the pollution of Cd in the sediments and soils of the Guishui River basin should be focused on in the future.

### 3.4. Health Risk Assessment of Potentially Toxic Elements Caused by Sediments

The accumulated concentrations of individual PTEs in fish during sediment disruption are shown in Table 3. Due to the lack of related data in the Guishui River, the TSS value of the Guanting Reservoir downstream of Guishui River was used for the calculation of the bioaccumulation assessment model in this study [42]. As shown in Table 3, Cu and Zn concentrations were highest and of the same magnitude, followed by Cr, V, Ni, and Pb; the lowest was Cd. The results were consistent with those of Gao et al. [18], who estimated the enrichment of PTEs in fish during water level fluctuations in the Three Gorges Reservoir. This indicated that the method in this study could successfully estimate the metal concentrations in fish without any real measurement in the fish.

In order to further assess non-carcinogenic risk for adults caused by sediments, the *HI_sediment_* values were calculated, as shown in Table 3. The total *HI_sediment_* was only 0.059, indicating that the local inhabitants should not experience adverse health effects from the studied PTEs. Meanwhile, it also indicates that there are no significant health risks to humans from the intake of individual PTEs through fish consumption during sediment disruption. The *HI_sediment_* values for the studied PTEs followed the order of Cr > Pb > Cu > V > Ni > Zn > Cd. Cr had the highest risk value (3.0 × 10^−^^2^), accounting for 51.08% of the total *HI_sediment_*.

### 3.5. Difference between Potentially Toxic Elements Exposure to Soils And Sediments

In order to accurately assess the difference of health risk between the PTEs exposure to soils and to sediments, the *HI_soil_* of the non-carcinogenic risk for adults were selected (as shown in Table 2) owing to the parameters in Equation (7) applied in the soils were same as those in the sediments. The result of *HI_soil_* and *HI_sediment_* shows that a significant difference exists in the order of *HI* value of PTEs. In addition, the *HI_sediment_* values of Cr, Ni, and Pb were similar to *HI_soil_*, but the *HI_sediment_* values of V, Cu, Zn, and Cd were one order of magnitude lower than those of *HI_soil_*. The difference may be due to the following reasons: 1) the desorption pathways were different. In sediment, the PTEs were desorbed from the sediment and absorbed by fish and then entered the humans via fish consumption [17]. This assessment model involved the process of food chain delivery, but the process was not involved in the pathway of exposure from soils; 2) there were discrepancies in the model assumptions. The health risk assessment model by fish consumption was built based on the equilibrium partitioning model and the kinetic model. Meanwhile, the estimated result was also affected by the water quality model [19]; however, the health risk assessment of PTE exposure from soil was based on direct contact between humans and soils, as related to human habits [17].

However, an interesting phenomenon was found which suggested that Cr exhibited a strong health risk for humans in the two exposure pathways. This is mainly related to the strong biotoxicity and high activity of Cr [43,44]. In particular, when Cr is present in the form of hexavalent chromium, it is not easily adsorbed by soil and is quickly released into the environment [44]. Thus, the bioavailability of PTEs should be focused on defining the guidelines or screening levels of PTE contaminants in the soils and sediments in the future. Overall, the health risk caused by PTEs in soils was slightly higher than that in sediment, and, the non- carcinogenic risk of Cr in the soil should also be paid greater attention in the future.

## 4. Conclusions

Overall, the soils and sediments were found to be at low risk, but Cd was identified as the main contaminant in the Guishui River basin according to the *I_geo_* and *RI.* However, different from the index evaluation results, Cd has a low risk to humans according to the health risk assessment model.

For the non-carcinogenic risk by PTE exposure from soils, ingestion was the main pathway and had the highest levels of exposure risk for children and adults, followed by dermal contact and inhalation. For the two subpopulations, children had greater health risks than adults did. The *HI_soil_* value of the studied PTEs for adults followed the order of As > Cr > V > Pb> Ni > Co > Cd > Cu > Zn, and the order was similar to that for children. Although the *HI_soil_* values of the studied PTEs were all lower than 1 (the safe level), As and Cr exhibited higher risks for humans via exposure to soil particles. For the carcinogenic risk effects caused by exposure to soils, the carcinogenic risk level for PTEs (As, Cd, Co, Cr, and Ni) were all lower than 10^−6^ magnitude. The carcinogenic risk level of Cr, however, was 1.86× 10^−^^7^ for adults and 6.59 × 10^−^^8^ for children, indicating that the carcinogenic risk of Cr must be paid more attention in the future.

For the non-carcinogenic risks caused by sediments, although the enrichment of Cu and Zn in fish was highest, Cr showed the highest non-carcinogenic risk value (*HI_sediment_* = 3.0 × 10^−^^2^). The *HI_sediment_* value of the studied PTEs followed the order of Cr > Pb > Cu > V > Ni > Zn > Cd. The HI_sediment_ values of PTEs were under the acceptable level, indicating that the local inhabitants should not experience adverse health effects by exposure from sediments. Overall, exposure to the two media (soil and sediment) has no adverse health effects on humans. Simultaneous risk assessment of the two exposure pathways (soil and sediment) will be helpful for the government to comprehensively identify PTE pollution at the scale of a river basin.

## Figures and Tables

**Table 1:** The concentrations of potentially toxic element in soils and sediments of Guishui River (mg/kg).

Location		As	Cd	Cr	Co	Cu	Ni	Pb	V	Zn
soil (*N* = 6)	Mix	5.41	0.10	36.27	7.13	12.54	15.43	18.90	51.45	46.12
	Max	10.60	0.27	58.56	11.96	29.86	27.84	36.01	78.08	162.05
	Mean	8.57	0.16	52.04	10.44	20.04	23.24	25.25	67.77	75.17
	Std	2.03	0.06	7.61	1.85	5.97	5.11	6.00	9.92	39.64
sediment (*N* = 5)	Mix	5.81	0.09	46.72	10.00	15.97	19.92	19.57	59.54	56.39
	Max	8.11	0.22	53.98	11.02	21.17	23.65	25.62	73.54	91.80
	Mean	6.81	0.14	50.45	10.48	17.95	21.78	22.42	66.95	66.76
	Std	1.02	0.06	3.58	0.46	2.30	1.55	2.48	5.77	16.91
Background values in soil of Beijing [23]		9.40	0.0534	66.70	15.00	23.10	28.20	24.70	77.40	97.20

Note: Std means Standard deviation.

**Table 2:** Carcinogenic and non-carcinogenic risks for individual potentially toxic elements and exposure pathway.

Parameter	As noncanc.	As canc.	Cd noncanc.	Cd canc.	Cr noncanc.	Cr canc.	Co noncanc.	Co canc.	Cu	Ni noncanc.	Ni canc.	V	Zn	Pb
C(mg/kg)	8.58	8.58	0.16	0.16	51.25		10.39	10.39	19.88	23.01	23.01	67.89	73.26	25.11
RfD Oral	3.0 × 10^−4^		1.0 × 10^−3^		3.0 × 10^−3^		2.0 × 10^−2^		4.0 × 10^−2^	2.0 × 10^−2^		9.0 × 10^−3^	3.0 × 10^−1^	3.5 × 10^−3^
RfD Inhal	3.01 × 10^−4^		1.0 × 10^−3^		2.86 × 10^−5^		5.71 × 10^−6^		4.02 × 10^−2^	2.06 × 10^−2^		7.0 × 10^−3^	3.0 × 10^−1^	3.52 × 10^−3^
RfD Dermal	1.23 × 10^−4^		1.0 × 10^−5^		6.0 × 10^−5^		1.6 × 10^−2^		1.2 × 10^−2^	5.4 × 10^−3^		7.0 × 10^−5^	6.0 × 10^−2^	5.25 × 10^−4^
Inhal.SF		4.3 × 10^−3^		6.3		42.0		9.8			8.4 × 10^−1^			
Children														
*HQ_ing_*	0.365		2.04 × 10^−3^		2.18 × 10^−1^		6.64 × 10^−3^		6.35 × 10^−3^	1.47 × 10^−2^		1.23 × 10^−1^	3.12 × 10^−3^	9.17 × 10^−2^
*HQ_inh_*	1.02 × 10^−5^		5.7 × 10^−8^		6.4 × 10^−4^		6.5 × 10^−4^		1.77 × 10^−7^	3.99 × 10^−7^		3.46 × 10^−6^	8.72 × 10^−8^	2.54 × 10^−6^
*HQ_dermal_*	4.28 × 10^−2^		3.26 × 10^−4^		1.75× 10^−2^		1.33× 10^−5^		3.39 × 10^−5^	8.72 × 10^−5^		1.98 × 10^−2^	2.5 × 10^−5^	9.78 × 10^−4^
*HI_soil_*	4.08 × 10^−1^		2.36 × 10^−3^		2.36 × 10^−1^		7.3 × 10^−3^		6.39 × 10^−3^	1.48 × 10^−2^		1.43 × 10^−1^	3.15 × 10^−3^	9.27 × 10^−2^
Carcinogenic risk		1.13 × 10^−12^		3.08 × 10^−11^		6.59 × 10^−8^		3.12 × 10^−9^			5.92 × 10^−10^			
Adults														
*HQ_ing_*	4.9 × 10^−2^		2.74 × 10^−4^		2.93 × 10^−2^		8.91 × 10^−4^		8.52 × 10^−4^	1.97 × 10^−3^		1.66 × 10^−2^	4.19 × 10^−4^	1.23 × 10^−2^
*HQ_inh_*	7.19 × 10^−6^		4.02 × 10^−8^		4.52 × 10^−4^		4.59 × 10^−4^		1.25 × 10^−7^	2.82 × 10^−7^		2.45× 10^−6^	6.16 × 10^−8^	1.8 × 10^−6^
*HQ_dermal_*	1.09 × 10^−1^		8.33 × 10^−4^		4.46 × 10^−2^		3.39 × 10^−5^		8.65 × 10^−5^	2.23 × 10^−4^		5.06 × 10^−2^	6.38 × 10^−5^	2.50 × 10^−3^
*HI_soil_*	1.58 × 10^−1^		1.11 × 10^−3^		7.43 × 10^−2^		1.38 × 10^−3^		9.39 × 10^−4^	2.19 × 10^−3^		6.73 × 10^−2^	4.83 × 10^−4^	1.48 × 10^−2^
Carcinogenic risk		3.19 × 10^−12^		8.69 × 10^−11^		1.86 × 10^−7^		8.81 × 10^−9^			1.67 × 10^−9^			

**Table 3:** The hazard index (HI) for individual potentially toxic elements from fish consumption.

Metal	V	Cr	Ni	Cu	Zn	Cd	Pb	Total
Concentration in fish (μg/g)	6.61 × 10^−2^	1.46 × 10^−1^	7.62 × 10^−2^	5.54 × 10^−1^	6.23× 10^−1^	3.98 × 10^−4^	5.93× 10^−2^	
RfDo (mg/kg·day) ^a^	7.0 × 10^−3^	3.0 × 10^−3^	2.0 × 10^−2^	4.0 × 10^−2^	3.0 × 10^−1^	1.0 × 10^−3^	3.5 × 10^−3^	
*HI_fish_*	5.83 × 10^−3^	3.0 × 10^−2^	2.35 × 10^−3^	8.55 × 10^−3^	1.28× 10^−3^	2.46 × 10^−4^	1.05 × 10^−2^	0.059
Contributions (%)	9.93	51.08	4.01	14.56	2.18	0.42	17.82	

^a^: Oral reference dose (RfDo).

## Supplementary Materials

The following are available online at https://www.mdpi.com/1660-4601/16/22/4539/s1, Table S1: The relative error and detection limit of each element in certified reference material of stream sediment (GSD–1a, GBW07301a), Table S2: Values of *I_geo_* and the pollution level, Table S3: Category of potential ecological risk factor, Table S4: The implication of each parameter in human health risk assessment model.
