# Supplementary material for: Simultaneous Health Risk Assessment of Potentially Toxic Elements in Soils and Sediments of the Guishui River Basin, Beijing
Source: Int J Environ Res Public Health. 2019 Nov 16;16(22):4539. doi: 10.3390/ijerph16224539 (PMC6888392; doi:10.3390/ijerph16224539)
Supplement: Supplementary file 1 [file ijerph-16-04539-s001.pdf]

Supplementary

Table S1. The relative error and detection limit of each element in certified reference material of stream sediment (GSD–1a, GBW07301a).

	**As**	**Cd**	**Cr**	**Co**	**Cu**	**Ni**	**Pb**	**V**	**Zn**
Measured value（mg/kg）	3.04	0.12	126.49	19.45	27.01	55.76	31.54	114.2	86.03
Reference value（mg/kg）	2.70	0.11	128	20	28	56	31	115	90
Relative error (%)	12.52	7.27	−1.18	−2.76	−3.54	−0.43	1.74	−0.70	−4.42
Detection limit (mg/kg)	0.12	0.05	0.11	0.03	0.08	0.06	0.09	0.08	0.67

Table S2. Values of I_{geo} and the pollution level.

I_{geo} ***rank***	I_{geo}	**Pollution Level**
0	<0	Uncontaminated
1	0-1	Uncontaminated to moderately contaminated
2	1-2	Moderately contaminated
3	2-3	Moderately to strongly contaminated
4	3-4	Strongly contaminated
5	4-5	Strongly to extremely strongly contaminated
6	>5	Extremely contaminated

Table S3. Category of potential ecological risk factor.

**Potential ecological risk factor (*EI*)**	***RI***	**Category**
$EI \leq 40$	$RI \leq 150$	Low ecological risk
$40 < EI \leq 80$	$150 < RI \leq 300$	Moderate ecological risk
$80 < EI \leq 160$	$300 < RI \leq 600$	Considerable ecological risk
$160 < EI \leq 320$	$600 < RI$	High ecological risk
$320 < EI$		Very high ecological risk

Table S4. The implication of each parameter in human health risk assessment model.

**Parameter**	**Implication**	**Unit**	**Reference value**	**Reference**
IngR	Ingestion rate	mg/d	Adults and children are 100 and 200, respectively	[1]
InhR	Inhalation rate	m^3/d	Adults and children are 20 and 7.6, respectively	[2]
EF	Exposure frequency	d/year	350	[3]
ED	Exposure duration	year	Adults and children are 24 and 6, respectively	[1]
SA	Exposed skin area	cm^2	Adults and children are 4350 and 1600, respectively	[3]
SL	Skin adherence factor	$mg/(cm^2 \cdot d)$	Adults and children are 0.7 and 0.2, respectively	[1]
ABS	Dermal absorption factor	unitless	0.001	[4,5]
PEF	Particle emission factor	m^3/kg	1.36E+09	[1]
BW	Average body weight	kg	Adults and children are 55.9 and 15, respectively	[3]
AT	Average time	d	for non-carcinogens, ED × 365 ; for carcinogens, 70 × 365;	[4,6]
C	exposure-point concentration	mg/kg	/	/
R_fDi	the daily oral reference dose	mg/kg•day	/	[5]
Di	the average daily intake by different exposure pathways	mg/kg•day	/	/
SF	slope factor of inhalation	unitless		[6]

Table S4. The implication of each parameter in bioaccumulation assessment model.

Parameter	Implication	Unit	Reference value	Reference
C_f	the HMs concentration in the fish	μg/g	/	
C_w	the HMs concentration in the aqueous phase	μg/L	/	
C_t	the total HMs concentration in the water	mg/L	/	
C_s	the total HMs concentration in the sediment	mg/kg	/	
TSS	the concentration of the total suspended solids (TSS)	mg/L	32	[7]
C_w	the HMs concentration in the water phase	mg/L	/	
C_p	the HMs concentration in solid (particular matter) phase	mg/kg	/	
K_d	the partition coefficient	L/kg	1.4×10^4	[8]
BCF	the bio-concentration factor of HMs in the fish			[9-12]

Note: "/" denote the value needs to be calculated; the values of BCF are V (75.2), Ni (264.5), Cr (200), Pb (200), Cd (200), Zn (700), Cu (2200) [9-12].

Reference

1. USEPA. Supplemental guidance for developing soil screening levels for Superfund sites. Office of Solid Waste and Emergency Response, 9355: 4–24 (OSWER). 2001.
2. Van den Berg R. Human exposure to soil contamination: a qualitative and quantitative analysis towards proposals for human toxicological intervention values. RIVM Report no. 725201011. Bilthoven, the Netherlands: National Institute of Public Health and Environmental Protection. 1995.
3. ESAG (Environmental site assessment guideline), 2009. DB11/T 656–2009. (In Chinese)
4. Ferreira-Baptista L.; De Miguel E. Geochemistry and risk assessment of street dust in Luanda, Angola: a tropical urban environment. Atmos. Environ. 2005, 39, 4501–4512.
5. Man, Y.B.; Sun, X.L.; Zhao, Y.G.; Lopez, B.N.; Chung, S.S.; Wu, S.C. Health risk assessment of abandoned agricultural soils based on heavy metal contents in Hong Kong, the world's most populated city. Environ. Int. 2010, 36, 570–576.
6. Zheng, N.; Liu, J.S.; Wang, Q.C.; Liang, Z.Z. Health risk assessment of heavy metal exposure to street dust in the zinc smelting area, Northeast of China. Sci. Total Environ. 2010, 408, 726–33.
7. Lei, X., Guo, Z.Q., Tian, Y., Xie, F., Qin,, J.X., 2013. Absorption characteristics of particulates and the CDOM in autumn in Guanting Reservoir. Journal of Lake Sciences, 2013, 25(6):883–891.
8. Hyoe, T.; Tatsuo, A.; Keiko, T.; Shigeo, U. Sediment–Water Distribution Coefficients of Stable Elements in Four Estuarine Areas in Japan. J. Nucl. Sci. Technol. 2010, 47(1), 111–122.
9. Gibbs, P. J.; Miskiewicz, A. G. Heavy metals in fish near a major primary treatment sewage plant outfall. Mar. Pollut. Bll. 1995, 30, 667–675.
10. Xu, Y. and Wang, W. X. Exposure and food chain transfer factor of Cd, Se, and Zn in a marine fish, Lutjnaus argentimaculatus. Mar. Ecol. Prog. Ser. 2002, 238, 173–186.
11. IAEA (International Atomic Energy Agency). Sediment distribution coefficients and concentration factors for biota in marine environment. Technical Report Series. Vienna. 2004, No. 422.
12. Wu, A.; Wei, J.; Sun, W.; Yuan, F.; Liu, Y. A study of biological enrichment of heavy metals to zebra fish with cultured water. J. Foshan University (Natural Sciences Edition). 2016, 34, 85–93.
